# The Landscape of mtDNA Modifications in Cancer: A Tale of Two Cities

**DOI:** 10.3389/fonc.2017.00262

**Published:** 2017-11-02

**Authors:** Kate L. Hertweck, Santanu Dasgupta

**Affiliations:** ^1^Department of Biology, The University of Texas at Tyler, Tyler, TX, United States; ^2^Department of Cellular and Molecular Biology, The University of Texas Health Science Center at Tyler, Tyler, TX, United States

**Keywords:** mitochondria, mitochondrial genome, mtDNA, DNA alteration, human cancer

## Abstract

Mitochondria from normal and cancerous cells represent a tale of two cities, wherein both execute similar processes but with different cellular and molecular effects. Given the number of reviews currently available which describe the functional implications of mitochondrial mutations in cancer, this article focuses on documenting current knowledge in the abundance and distribution of somatic mitochondrial mutations, followed by elucidation of processes which affect the fate of mutations in cancer cells. The conclusion includes an overview of translational implications for mtDNA mutations, as well as recommendations for future research uniting mitochondrial variants and tumorigenesis.

## Introduction

In the best of times, mitochondria are cytoplasmic organelles that operate as power generators. In a normally functioning cell, mitochondria provide the “energy currency,” which allows cells to carry out normal metabolic function. In the worst of times, mitochondrial dysfunction may contribute to an array of human health disorders, including multiple types of cancer (1, 2). Indeed, mitochondria represent a microcosmic tale of two cities, wherein the dynamic processes governing the organization and response of the organelle differ between normal and cancerous cells. The specific mechanisms through which mitochondria affect cancer progression represent a complex intersection between changes in physiological function and DNA sequence, with concomitant feedback among these processes.

Mitochondria occupy a canonical cellular role as “the powerhouses of the cell,” synthesizing adenosine triphosphate (ATP) through the process of oxidative phosphorylation. Additional functions provided by mitochondria range from complementary roles in cellular metabolism to more far-ranging relationships with inflammation and programmed cell death (3). Cancer cells, however, are characterized by high levels of both energy requirements and proliferation, meaning mitochondria may often play a central role in cancer induction and progression (4, 5).

In addition to oxidative phosphorylation, other mitochondrial functions have been implicated in cancer formation and progression (6). In the 1950s, Otto Warburg identified mitochondria as a contributor to aerobic glycolysis, in which glucose ferments to pyruvate in the presence of oxygen (7, 8). Although physiological aberrations leading to aerobic glycolysis do not appear to impair mitochondrial function, they are a footprint in the roadmap of malignant transformation (9). Additionally, by serving as executors of programmed cell death, mitochondria have the capacity to contribute to avoidance of apoptosis, allowing cancer cells to continue proliferating (10). Several recent reviews have thoroughly documented mitochondrial dynamics in relation to cancer (11, 12). Through various mechanisms, these cellular functions interact with genetic alterations in the mitochondria to provide a feedback mechanism contributing to cancer emergence and progression (3, 4).

**Figure 1:**
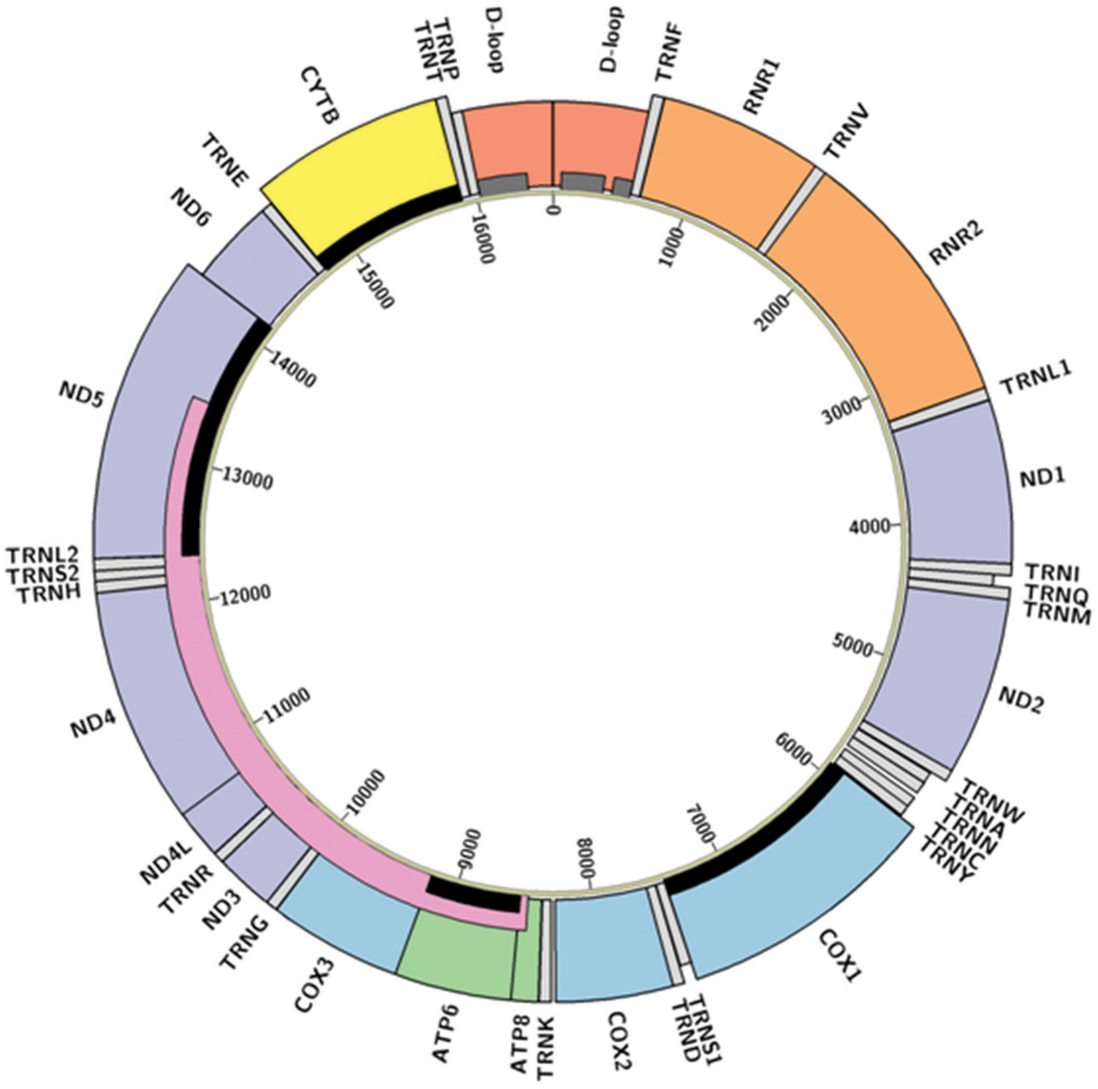
Structure of the human mitochondrial genome. Colored boxes represent locations of D-loop (red), transfer RNAs (light gray), ribosomal RNAs (orange), Complex I NADH dehydrogenase genes (purple), Complex III cytochrome c reductase gene (yellow), Complex IV cytochrome c oxidase genes (blue), and Complex V adenosine triphosphate synthase genes (green). Boxes appearing shorter represent antisense transcripts; others are sense transcripts. Hypervariable regions (16) are represented by dark gray bars, the “common deletion” (17) as a pink bar, and the genes described in Abundance and Distribution of Mitochondrial Mutations as relevant to cancer are highlighted with black bars. Diagram created in Circos (18) from GenBank accession NC_012920 (revised Cambridge Reference Sequence); code available at https://github.com/k8hertweck/mt_genome_viz.

Mitochondria are the descendants of a billion-year-old symbiosis between bacterial precursors and pre-eukaryotic cells (13). Along with their autonomous bio-energetic sister organelles, chloroplasts, they retain specialized anatomical structures, self-contained haploid genomes, and corresponding regulatory processes. Specialization of these organelles has resulted in much of their genetic material being transferred to the nuclear genome, with a redox regulatory system retained in the organelle to preserve function (14). In humans, each mitochondrion holds a circular, intron-free, double-stranded genome that is only 16.5 kb in size, encompassing 13 protein-coding genes, 22 transfer RNAs (tRNAs), and 2 ribosomal RNAs (rRNAs) (Figure 1). Despite this small size, mutations to the mitochondrial genome (mtDNA) have been associated with multiple types of cancer during the formation, growth, and metastasis of tumor cells (15). However, the specific mechanisms through which these mtDNA changes alter physiological processes remain unclear (9). We contend that this lack of clarity is partially derived from a lack of understanding about general patterns in mtDNA mutations related to cancer, which this review seeks to synthesize.

In the nearly two decades, since the first report of somatic mtDNA mutation associated with human cancer (19), mtDNA alterations associated with cancer formation and progression have been documented throughout the mitochondrial genome and in many different cancer types. This review focuses on the mutational landscape of the mtDNA in cancer, including the type, distribution, and frequency of mitochondrial mutations. We then review clinical translation of mtDNA mutations before identifying future directions for research on the mtDNA cancer genome.

## The Mitochondrial Genome’s Mutational Landscape

Complete genome sequencing of diverse cancer types from many individuals has revealed overwhelming evidence of the influence of nuclear mutations on cancer susceptibility, formation, and progression (20). The origin and impact of mtDNA alterations remain unclear, however, with large-scale studies across multiple tumor types providing scant evidence generally connecting mitochondrial mutations with formation and spread of tumors (15, 21). On the other hand, focused studies on specific tumor types suggest closer relationships between mtDNA alterations and tumorigenesis (22). Ultimately, genomic instability driving mutation accumulation is considered a genetic hallmark of cancer progression (23), and mtDNA alterations are emerging as an essential but complex feature of this multifaceted process.

### Abundance and Distribution of Mitochondrial Mutations

The difficulty in assessing the contribution of mtDNA mutations to tumorigenesis is largely related to nuances associated with genetics of the organellar genome. Most importantly, the proximity of reactive oxidative species (ROS) formed during normal metabolic events increases risk of mtDNA perturbation and instability (3). The effect of ROS on changes to mtDNA is supported by a general increase in some cancers of transitions at purines (19). This risk to DNA damage, coupled with fewer repair mechanisms relative to the nuclear genome, results in an mtDNA mutation rate at least an order of magnitude higher than the nuclear genome (24). Empirical evidence suggests that endogenous mutational processes are much more influential in the mtDNA mutation rate, as opposed to exogenous carcinogens like environmental chemical and ultraviolet light (15). Two additional characteristics are relevant to interpret the abundance and distribution of variants in mtDNA. First, mitochondria (and mitochondrial genomes) are present in hundreds or thousands of copies per cell. Second, mtDNA exhibits matrilineal inheritance in humans, resulting in a single mtDNA haplotype per individual (but see Heteroplasmy for the effects of multiple copies).

Both germline and somatic variants are relevant to interpreting genetic alterations in mtDNA. Germline mtDNA mutations are heritable from mother to offspring and are constitutively found throughout the body of the offspring. Somatic mutations, on the other hand, cannot be inherited by offspring but can be found in subsequently proliferating populations of cells. Germline mtDNA variants are useful for assigning individuals to certain haplogroups (25), which can then be related to ancestral matrilineal relationships (16). Although haplogrouping is primarily used for identifying relationships among individuals and populations, some haplogroups possess sequence variants that also contribute to cancer susceptibility (26–28). Moreover, some somatic mutations may represent haplogroup conversion (29), complicating interpretability of mtDNA data.

The basic landscape of somatic mutation across the mitochondrial genome of cancer reflects more general patterns in germline mutational processes. Two broad assessments of many cancer types (15, 21) noted replicative strand bias in mtDNA mutations associated with cancer. Although this latter trend differs from that found in the nuclear cancer genome, it does recapitulate germline patterns shaping primate mtDNA mutations. In the context of mammalian mitochondrial genomes, human mtDNA in general contains mutation hotspots in both rRNA and protein-coding genes, representing synonymous, non-synonymous, and non-coding sites, with minimal changes in tRNAs (30). This relative frequency of mutation in various sequence types is reflected in whole-genome analysis of somatic mutations related to cancer (31, 32). Signatures of cancer mutations reported from the nuclear and mitochondrial are both heterogeneous across tumor types (15, 21). Moreover, the proportion of individual cases containing somatic mutations varying from 13 to 63% depending on the type of cancer (33) and mtDNA variants may be present across cancer types or present only in one type of tumor (34). A comparison of distribution and types of somatic mtDNA mutations from exemplar literature is reviewed in Lee et al. (35); summaries below represent patterns emerging as relevant across cancer types.

Multiple mitochondrial genes have documented somatic mutations which may be implicated in tumor formation (Table 1; Figure 1). Across cancer types, somatic mtDNA alternations are enriched for non-synonymous variants compared to synonymous variants (32, 33). Protein-coding genes found in the mitochondria belong to four different complexes of the mitochondrial respiratory chain (Figure 1). Complex I (NADH dehydrogenase) is represented by seven mtDNA genes (*ND1-6*, including *ND4L*); this complex most frequently contains variants related to tumorigenesis (36). *ND5*, for example, is enriched for somatic mutations (31, 33, 37), which may alter tumor progression (38). Complex III, of which *CYTB* is the only gene encoded by mtDNA, contains fewer documented somatic variants. The exception is bladder cancer, in which this complex is significantly more affected than other complexes (39) and a 7 amino acid deletion detected in natural populations is experimentally associated with bladder tumor growth (22, 40). Complex IV (cytochrome c oxidase) includes three genes encoded in the mitochondria (*COX1-3*); mutations in *COX1* associated with colorectal cancer may eliminate expression or decrease the efficiency of respiratory chain (41). Finally, Complex V (ATP synthase) has two mitochondrially encoded genes (*ATP6* and *ATP8*). *ATP6* appeared more susceptible to mutation than *ATP8* in breast cancer patients, which may reflect changes in energy metabolism among cancer cells (42). Across these protein-coding genes, alterations to Complexes I and IV appear to be the most influential in inducing tumorigenesis (12).

**Table 1:** Mitochondrial mutations associated with cancer summarized by tumor type.

Tumor type	Citations	D-loop variants	Variants occurring frequently in genes
Bladder	(39, 43–45)	Subs and indels in 57% of cases	Subs and indels in ribosomal RNA (rRNA) and protein-coding genes (*ND3, ND4, ND5*, and *CYTB*)
Breast	Reviewed by Salgado et al. (29) and Yadav and Chandra (46) (31, 37, 42, 44, 47, 48)	Subs and indels in 16–43% of cases; D310 instability in 29% of cases	More frequent mutations in *ATP6* than *ATP8, ND5* mutations in 9% of cases; *ND3* (G10398A) may be important (but controversial)
Colorectal	Reviewed by Skonieczna et al. (33, 49)	Subs and indels in 7–40% of cases; D310 instability in 23–44% of cases	Low frequency of mutation in protein-coding genes and transfer RNAs (tRNAs) with higher frequency in rRNAs; synonymous and non-synonymous subs across all genes; 56% of cases with non-synonymous mutations
Gastric	Reviewed by Lee et al. (35)	Subs and indels in 4–48% of cases	Synonymous and non-synonymous subs in protein-coding genes; subs and indels in tRNAs
Head and neck	(32, 43, 44, 50, 51)	Subs and indels in 21–37% of cases, majority associated with D310 instability	Synonymous and non-synonymous subs in protein-coding genes (*ND4, ND5*, and Complex IV); subs and indels in tRNAs and rRNAs
Lung	(43, 44, 52–56)	Subs and indels in 23–35% of cases, D310 instability in 20% of cases	Synonymous and non-synonymous subs in protein-coding genes (enriched in Complex I); subs and indels in tRNAs and rRNAs
Ovarian	(33, 44, 57–59)	Subs and indels in 20–57% of cases; D310 instability may occur rarely	Synonymous and non-synonymous subs in protein-coding genes (*ND4*); subs in rRNA
Prostate	(44, 60–63)	Few mutations in D-loop, D310 instability in 0% of cases	Synonymous and non-synonymous subs in protein-coding genes (*COI*, Complex I); subs and indels in rRNA and tRNA; mutational load increases with metastasis

*Cancers listed represent most well-characterized mtDNA variation. Studies listed focus largely on common patterns in somatic single nucleotide variants, including substitutions (subs) and small insertions/deletions (indels, including homopolymer changes). D310 is C-homopolymer stretch located near bp 310 of D-loop. Several studies have assessed mtDNA mutations across multiple types of cancer (15, 21); Lee et al. (35) reviewed location and type of somatic mitochondrial DNA mutations in various types of human cancer*.

The mitochondrial genome includes 22 tRNAs, comprising a relatively small proportion of the mtDNA nucleotide sequence. Somatic mutations in tRNA are not frequently documented in association with human cancer (Table 1), although they are commonly implicated in a variety of other primary respiratory chain disorders (2). Of the relatively few tRNA mutations associated with cancer, they appear to represent alteration to secondary structures (21) and variants in stem and loop regions may result in instability and altered mitochondrial function (31). Similarly, mutations in the two mitochondrially encoded rRNA genes are more likely to result in dramatically deleterious effects than changes to protein-coding genes (2); thus, modifications to rRNA are even less common (Table 1).

While genic regions of the mitochondrial genome are a logical target for assessing cancer mutations, hypervariable (HV) sites in the non-coding human mtDNA control region also represent general mutational hotspots. Both germline and somatic mutations in mtDNA occur preferentially in two parts of this region, HV1 and HV2 (64). Cancer research has focused intensely on genetic variants in a large portion of the control region called the D (displacement) loop. The D-loop is a subset of the non-coding region found in many animal mtDNAs and is formed by incorporation of a third linear strand of DNA. Given the association between the control region and mitochondrial replication and transcription, mutations in the D-loop may influence the copy number and regulation of mitochondria (65). In the context of cancer, D-loop mutations are arguably the most well-studied of all mtDNA cancer variants (Table 1). This region possesses high enough rates of change to assess progression and proliferation of cell lineages (47). Mutations in the D-loop are also more frequent in later stages of cancer (54), and higher numbers of somatic D-loop mutations have been linked to poor prognosis in breast cancer (66). Despite possible implications D-loop mutations may have for mitochondrial function in cancer, it is unclear whether these variants are a causal or simply associated phenomenon (but see Natural Selection on Genetic Variants in Tumor Formation) (47, 66, 67).

### Natural Selection on Genetic Variants in Tumor Formation

The conventional view of cancer as a genetic disease is fueled by somatic mutation theory, which describes cancer as originating in the nuclear genome through a handful of “driver gene” mutations (68). The theory describes subsequent mutation accumulation throughout the genome as a result of these variants in oncogenes, resulting in the array of characteristics defining tumor development (23). Somatic mutation theory, however, is difficult to reconcile with documented mtDNA variants associated with cancer (69). Increasing evidence implicating mitochondria in cancer risk, emergence, and progression contributes to a growing acknowledgment of cancer as a mitochondrial metabolic disease. This evidence derives from various processes involving natural selection, heteroplasmy, and the combined effects of genetic alterations across the genome.

Following the emergence of mutations in individual mitochondrial genomes, variants are subsequently subjected to a variety of molecular, cellular, and population level processes (70). Tumorigenesis is ultimately dictated by evolutionary processes, wherein mutations within the genome emerge and are then subjected to natural selection and/or genetic drift. The effects of selection are traditionally separated into two categories in cancer research: first, purifying (negative) selection, in which deleterious alleles are removed from the population, and second, positive selection, whereby advantageous alleles increase in frequency in a population (perhaps to fixation). Alternatively, mutations may be neutral and not subject to selective pressures but still proliferate stochastically through drift.

Numerous datasets and approaches have been applied to test for selection in mtDNA mutations associated with cancer. Throughout human history, germline mtDNA mutations in genes exhibit negative selection (71). Evidence from a large sample of mitochondrial genomes suggests that mutations are subject to similar selective constraints, regardless of whether they arise in tumor or normal cells (72). Deleterious mutations tend to be selected against (9), but some normal tissues conversely exhibit positive selection on mtDNA somatic mutations. In liver, for example, positive selection reduced mitochondrial function to decrease damage ensuing from byproducts of metabolism (73). In terms of selection on mutations related to cancer formation, an assessment of somatic mtDNA mutations across oncocytic tumor types indicates that variants associated with cancer are indistinguishable from random (74). However, there is a correlation between the number of somatic mtDNA mutations and patient survival in breast cancer, with evidence for both positive and relaxed negative selection for somatic missense mutations (31). Surveys of mtDNA alterations in bone metastases of prostate tumors revealed statistically greater variation than both primary tumors and soft tissue metastases (62). These studies, though seemingly contradictory, cumulatively highlight two points in the effect of selection on mtDNA mutations in cancer: first, the time scale and differentiation of somatic from germline mutations matters, and second, patterns of selection may be tissue (and therefore tumor) specific.

### Mitochondrial Structural Variation Associated With Cancer

Single-nucleotide substitutions receive much of the focus in cancer studies given their possible connections to protein changes. However, genomic instability encompasses a wide array of additional variants as well, such as structural variations (e.g., duplications and deletions). A meta-analysis of such structural changes in mtDNA associated with human disease noted that mtDNA duplications are not reported in tumors, although it is unclear whether they are less frequent than in other diseases or simple not often assessed (75). The nature of structural variants in the mitochondria ranges from small (single nucleotides) to large (hundreds to thousands of bases) (Table 2).

**Table 2:** Mitochondrial structural variants summarized by tumor type.

Tumor type	Citations	Structural variant
Bladder	(43)	21 bp deletion in *CYTB*
Breast	Reviewed by Salgado et al. (29) and Yadav and Chandra (46, 76)	4977 deletion more common in cancer than normal tissue (but this is controversial)
Colorectal	(77, 78)	4977 deletion more and less common in tumor than normal tissue (conflicting results); novel deletion frequency varies with ethnicity
Gastric	Reviewed by Lee et al. (35)	4977 deletion more common in cancer than normal tissue; other deletions and duplications in D-loop frequent in cancer
Head and neck	(79)	4977 deletion more common in cancer than normal tissue

*Cancers listed represent those with the most well-characterized mtDNA variation. Included studies focus on large somatic structural variants and mitochondrial microsatellite instability; see Table 1 for single base pair insertions and homopolymer slippage. 4977 deletion refers to the “common deletion” spanning five tRNA genes and seven protein-coding genes (17) (Figure 1)*.

In terms of small structural variants, microsatellite instability (MSI) refers to changes in the number of short (1–5 bp) tandem repeats (sometimes also referred to as homopolymer slippage). Although variants of this type (especially from slippage associated with mononucleotide repeats) are often discussed with substitutions in cancer literature, they are presented here with other structural variants given the nature of the genetic variation. High levels of nuclear MSI throughout the nuclear genome have been associated with tumorigenesis in some types of cancer (29, 80). Frequency of this hypermutator phenotype within and across cancer types, however, is variable, and nuclear MSI is not associated with mitochondrial MSI (81) or mtDNA mutations in general (82).

Microsatellite instability in mtDNA is perhaps best studied in the D-loop, which reflects a finding that deletions involving the D-loop occur significantly more frequently in tumors than in other mitochondrial diseases (75). More specifically, the D310 homopolymeric (mononucleotide) C stretch possesses variants in ca. 22% of tumors across cancer types, although this estimate varies widely among and within tumor types and may be present in non-cancerous cells as well (44, 83, 84). Although this repeat is the most unstable in the D-loop, the frequency of changes at this locus and other mtDNA microsatellites is uncorrelated with and less frequent than nuclear MSI (81). Given this section of the D-loop is responsible for replication of the mtDNA heavy strand, Bragoszewski et al. (58) tested the relationship between D310 slippage with clinical status and expression of mtDNA genes. Although they found no relationship between the mononucleotide variant and clinical status (58), an earlier study suggested as association between a separate 50 bp somatic deletion in the D-loop with the location of gastric tumor origin (85). These findings cumulatively underscore the heterogeneity of responses to mitochondrial D-loop changes regarding cancer formation and progression.

In terms of larger structural variants within involving genes, a 21-bp deletion of *CYTB* was noted in bladder cancer patients. Overexpression of the mutated *CYTB* gene resulted in increased cell growth, which suggests a mechanistic relationship to tumorigenesis (40). An even larger deletion involving 4,977 bp (the “common deletion”) spanning five tRNA genes and seven protein-coding genes is one of the most frequently observed mitochondrial deletions in human tissues (17) and may be associated with endrogen receptor-positive breast cancer and lymph node metastasis (76). Meta-analysis suggests that the deletion is frequent in cancer but is selected against in some cancer tissues (86).

The most extreme example of structural rearrangements associated with cancer comes from recent documentation of somatic mtDNA transfers to the nuclear genome, which occur at a similar rate to interchromosomal rearrangements in the nucleus (87). The sequences involved in these transfers spanned the mitochondrial genome, but mitochondrial breakpoints were enriched near the heavy strand origin of replication for the heavy strand (in the D-loop), which may ultimately affect the number of mitochondria present in the cell.

### Mitochondrial Copy Number

In a normal cell, mitochondria (and genomes contained therein) occur in high copy number. Comparisons of mtDNA content in 15 cancer types with normal adjacent cells revealed that seven had decreased mtDNA copies in tumor cells (bladder, breast, esophageal, head/neck squamous cell, kidney, and liver), one increased (lung adenocarcinoma), and seven had no difference from normal mtDNA content (colorectal, kidney, pancreatic, prostate, stomach, thyroid, and uterine) (88). These patterns, however, are not entirely consistent with studies of individual cancer, which may reflect tumor-specific patterns (29) (Table 3). For example, more focused studies found that thyroid (89) and pancreatic (90) cancer cells are generally enriched for mitochondria, which may be a consequence of cell compensation for defective oxidative phosphorylation and lower ATP production per mitochondria and contribute to increased cancer risk. On the other hand, hepatocellular carcinoma cells are mitochondrially depauperate (91). The causal mechanism behind decreased mitochondrial copy numbers may be related to D-loop mutations, since this region mediates mtDNA replication (54).

**Table 3:** mtDNA copy number variation documented by tumor type.

Tumor type	Citations	mtDNA content relative to normal tissue (% of cases)
Bladder	(88)	Decrease
Breast	Reviewed by Salgado et al. (29) and Yadav and Chandra (46) (31, 88);	Decrease (71–88%)
Colorectal	(54, 77, 88, 92)	Decrease (28%) or no difference (conflicting results; may be tumor-specific); decrease related to presence of 4977 deletion
Gastric	Reviewed by Lee et al. (35)	Decrease
Head and neck	(79, 88)	Decrease and increase (conflicting results)
Lung	(54–56, 88)	Decrease and increase (conflicting results)
Prostate	(88, 93)	Increase (78%) and no change (conflicting results, may be associated with increased distributional variance in cancer cells)

*Cancers listed represent those with the most well-characterized mtDNA variation. 4977 deletion refers to the “common deletion” spanning five tRNA genes and seven protein-coding genes (17). Comparisons of mitochondrial copy number between normal and cancer cells in 22 tumor types is assessed in Reznik et al. (88)*.

### Heteroplasmy

The coexistence of multiple copies of the mitochondrial genome in each cell provides the basis for unique genetic patterns relative to the nuclear genome. Locations in the mitochondria which are identical throughout a cell (or sample) are called homoplasmic. Heteroplasmic sites represent mtDNA locations where multiple haplotypes are present in the same cell, which may exist through heteroplasmy in progenitor cells or *de novo* mutation. Although normal tissue is assumed to possess homogenous mtDNA, the frequency of heteroplasmic variants differs among even normal tissues in the same individual (73, 94) with additional homo- and heteroplasmic mutations in cancer cells (95). Moreover, mutations initially identified as putatively somatic may represent low-level heteroplasmies from germline tissue (94). Kloss-Brandstatter et al. (32) found oral cancer tissue to be enriched for non-synonymous heteroplasmic variants. Moreover, low-level heteroplasmy was more frequent in benign tissue than tumors and occurrence of heteroplasmy increased with metastasis.

Heteroplasmy represents one specific type of genetic variation, which is also intricately tied to the processes driving genetic variation in the mitochondrial genome. In pancreatic cancer, contributors to cancer risk include the following three types of alternations: variants common among humans, variants that are rare among human populations, and singletons, or variants that are found only in a single individual (27). The effect of heteroplasmy is that multiple mitochondrial genotypes are expressed simultaneously, and the ratios of common, rare, and singleton heteroplasmic variants dynamically adjust with subsequent physiological and genetic changes (96). Given the presence of purifying selection, how do such diverse variants persist in populations of cells and humans? Low-frequency (rare or singleton) heteroplasmy may allow potentially pathogenic mutations to persist because of the simultaneous functioning of unmutated copies (97). Late-onset mitochondrial disorders may continue to occur through fixation of less-severe variants, which manage to escape germline selection (98). Computational modeling suggests that selection is not required for low-frequency heteroplasmic mutations to reach fixation, indicating drift as a possible mechanism for this phenomenon (99).

### Nuclear-Encoded Alterations Affecting Mitochondria in Cancer

Mitochondrial dynamics and their associated genetics result in evolutionary interactions driving emergence and proliferation of mutations. An extra layer of complexity arises through interactions with nuclear genes. Of the thousand proteins comprising the mitochondrial proteome, only a small percentage are encoded by mtDNA (100). The rest of these proteins arise from genes located in the nuclear genome but which encode for mitochondrial replication and expression (3). Mutations to such genes may result in direct effects to instability and copy number of mtDNA, and nuclear-encoded genes may influence mitochondrial function in ways that also influence cancer formation and progression.

A limited number of nuclear-encoded genes have been hypothesized to alter the mitochondrial genome itself. For example, nuclear-encoded mitochondrial transcription factor A (TFAM) truncation arising from frameshift mutations in a coding mononucleotide repeat of the gene in colorectal cancer cell lines resulted in mitochondrial instability and reduced mitochondrial copy number (101). Somatic mutations in nuclear-encoded polymerase gamma, on the other hand, did not appear to increase mtDNA mutations, although they did result in mitochondrial dysfunction through a decrease in mtDNA content (102).

In addition to directly affecting mtDNA, genes from outside the mitochondria can have dramatic effects on mitochondrial function and dynamics. More specifically, nuclear-encoded genes, which contribute to mitochondrial functions like oxidative phosphorylation, can possess mutations, which increase cancer risk, produce oncogenic metabolites, or even initiate tumorigenesis (9, 103). *TP53* (tumor protein p53), for example, is a nuclear-encoded tumor suppressor which acts through cell cycle regulation and other cellular mechanisms to regulate mitochondrial metabolism. Some missense *TP53* mutations result in proteins, which continue to successfully promote normal or cancerous cell survival (104). Vyas et al. (5) highlighted additional genes involved in the following mitochondrial dynamics: biogenesis, mitophagy, fission/fusion, cell death, oxidative stress, metabolism, and signaling. While an overview of specific genes involved in each pathway represented by these functions is outside the scope of this review, this highlights the importance of understanding the nuclear landscape of expression changes concurrently with that of the mitochondrial genome itself.

### Integration of Evolutionary Processes with Types of mtDNA Variation

The review thus far has focused on individual types of variation and how evolutionary processes may affect their fates. In living cells, however, it is the interactions among these variants and evolutionary processes which dictate the development and progression of tumors. Assessment of mutational patterns across a large sample of tumors confirms that missense mutations tend to be selectively neutral and subjected to homoplasy through genetic drift. In contrast, structural variants which truncate proteins are subject to purifying selection but can persist through heteroplasmy because of compensation by fully functional mitochondria (15). Because pathogenic mtDNA variants are subjected to purifying selection, decreasing mtDNA content limits tumor formation because of the remove of this compensation. Evidence of this balance comes from oncocytomas, which are relatively rare and characterized by excessive mutation accumulation but ultimately benign (9).

Cytoplasmic hybrids, or cybrids, represent a powerful tool to test the evolutionary dynamics of mtDNA mutations empirically. A cybrid study in multiple human cancer lines tested whether mtDNA instability influences mtDNA depletion (105). The results indicated that these phenomena occur independently and that decreased mtDNA copy number does not contribute to the shift from heteroplasmic to homoplasmic. A separate cybrid study in which normal mitochondria were inserted into cancerous cells indicated that interactions among cancerous and non-cancerous mitochondria can reverse the physiological reactions associated with cancer cells, even in the context of a cancerous nuclear genome (106). These studies cumulatively highlight the importance of mitochondria in mediating the formation and progression of tumors. Moreover, the fate of mutations in mtDNA is a function of multiple, sometimes contradictory, processes and is largely contingent on individual tumors or cancer types.

## Continuing and Future Research on the Cancer Mitochondrial Genome

Studying mtDNA mutations related to cancer informs our understanding of a broad variety of concepts in basic science, such as mitochondrial tRNA function (21) and mechanisms of mtDNA replication (15). These findings, however, are generally pursued with the intention of clinical translation. This section summarizes clinical translations of mtDNA mutation studies, methodological recommendations for pursuing such research, and the emerging area of mitochondrial epigenetics.

### Clinical Translations of Mitochondrial Alterations in Cancer

The relationship between germline mtDNA mutations and cancer risk is addressed above (section Abundance and Distribution of Mitochondrial Mutations), but mtDNA variants can also inform cancer detection, treatment, and prognosis. Because mitochondria occur in high copy number in cells and are clonal in nature, they possess an innate capacity to aid in detection and diagnosis of some cancer types (43). The use of mtDNA as a biomarker is not limited to tumors, however, as mtDNA variants related to cancer can also be detected in minimally invasive bodily fluids (107), such as the use of urine to detect bladder cancer (39). Additional examples include the use of serum and nipple fluid aspirate for diagnosis of colorectal and breast cancer, respectively (108, 109).

Numerous studies have declared the utility of targeting the mitochondria to treat cancer (3, 9, 103, 110–112), even stating that “understanding mechanisms of mitochondrial function during tumorigenesis will be critical for the next generation of cancer therapeutics” (5). Given that apoptosis is a main contributor to the reduction of tumor cells and cell death response is controlled by the mitochondria, it logically follows that mtDNA mutations may alter responses to cancer therapy (113). In fact, a well-documented case study of a somatic mutation in *ND4* in serous ovarian cancer may be associated with chemoresistance (59) and D-loop mutations are correlated with resistance to chemotherapy in colorectal cancer patients (114). Decreased mtDNA content is connected with improved prognosis in breast cancer patients receiving anthracycline-based chemotherapy (115). In stark, contrast stand claims that levels of both mitochondrial gene expression and mtDNA mutations have limited clinical relevance, such as for ovarian cancer (58). A combination of individualized metabolic processes associated with myriad variation in both the nuclear and mitochondrial genomes prompts additional research into the use of personalized medicine in treating cancer (4).

### Methodological Recommendations for Future mtDNA Cancer Research

As knowledge of mtDNA mutations associated with cancer continues to grow, it is incumbent on researchers to manage these data and target the most appropriate directions for future research. The wide availability of massively parallel (next-generation) sequencing to obtain complete mtDNA sequences has increased the capacity to assess mutation across mitochondrial genome. With this technology, however, comes a corresponding need to appropriately design experiments and evaluate the robustness of results. A number of potential pitfalls exist in assessing mtDNA variants associated with cancer, including errors with labwork, misdocumentation, and incomplete referencing of previously published information (116).

The first issue concerns obtaining an appropriate representation of mtDNA mutations to assess. For instance, the use of paired tissue samples (tumor and normal) is essential for identifying somatic and germline variants. Additionally, Fang et al. (117) advocate use of the same material for diagnosis and DNA analysis (e.g., microdissect tissue from slides used for pathology). In terms of genomic sampling, the potential transfer of mitochondrial sequences to the nuclear genome may require differentiating nuclear copies of mitochondrial genes and pseudogenes (117), depending on the molecular sampling strategy. Many studies have assessed the sensitivity and accuracy of variant analysis (118). Although it is possible to obtain many putative variants, application of computational stringency filters may dramatically limit the number of variants identified (119). These filters are especially important when attempting to assess levels of heteroplasmy, particularly of low-frequency variants (120). Multiple tools have been suggested to increase robustness and interpretability of results. Reanalysis of previously published mtDNA results suggests that the use of median networks can identify potential problems with sequencing and/or documentation (121). It is becoming more commonplace to analyze variants using a phylogenetic approach and to search multiple databases to differentiate novel from known (rare and common) variants (122, 123). Finally, using the revised Cambridge Reference Sequence to standardize notation and minimize errors in interpretation (124).

Once mtDNA variants have been identified, the next goal is connecting them to potential functions; whether most variants are a cause or consequence of tumorigenesis remains uncertain (9). Identification of somatic mutations is not an assurance of functional relationships with cancer; for example, a 2006 assessment of tumor-specific mtDNA somatic mutations found 72% were variants also found in the general population (125). Furthermore, Covarrubias et al. (126) identified epistatic interactions between mitochondrial variants involved in risk of breast cancer. Subsequent reanalysis using a phylogenetic framework and more robust statistics indicated this relationship may be spurious (127). However, experimental evidence from cybrid studies in mice suggested that one of these variants (G10398A) allows cells to metastasize and resist apoptosis (128), indicating importance of functional studies to complement genomic characterization.

The considerations above suggest research topics worthy of future work. First, somatic mtDNA mutations clearly need to be assessed in the context of potential function, as this may refute or implicate mutations in cancer progression. For example, Ishikawa et al. (129) used cybrids to determine that enhanced glycolysis caused by mtDNA mutations does not actually induce metastasis. Cybrid studies introducing *COI* mutations in prostate cancers, however, confirmed that *COI* mutations dramatically increased tumor growth compared to wild type (60). Second, given the relationship between mitochondrial function and numerous loci in the nuclear genome, the significance of mtDNA mutations will be clearer if assessed in conjunction with variation in the nuclear genome (130) and adjusting for population substructure while assessing such epistatic interactions (103). A corresponding goal is identifying the extent to which a mutator phenotype (131) may contribute to mtDNA changes. Finally, effective simulation and modeling of mutational processes associated with cancer requires adequate knowledge of both biological function and experimental data and may need to be assessed for specific types of cancer independently (132). In general, the most promising areas for further research lie in the integration of multiple data types, spanning sequence variants, gene expression, and consequences on phenotype.

### The Mitochondria, Epigenetics, and Cancer

An emerging focus of cancer mitochondrial research is mitoepigenetics, which describes not only the epigenetic regulation (e.g., modifications to gene expression) of the mitochondrial genome but also corresponding interactions with the nuclear genome (133). Mitochondrial methylation patterns vary among normal human tissues (134). Moreover, whole-genome methylation is associated with a variety of human diseases, including cancer (135). These basic characteristics lead to an expectation that mtDNA epigenetic patterns could promote cancer progression. The influence of mitochondrial abundance on global patterns of gene expression (136) adds credence to a possible connection between methylation and mtDNA copy number (133).

Moreover, Minocherhomji et al. (137) propose a mechanism for this relationship: a mitochondrial damage checkpoint (which balances apoptotic signaling) may activate to repair damaged mitochondria. If signaling occurs between the nucleus and mitochondria, the mitocheckpoint could potentially alter epigenetic patterns and genomic stability. These lines of evidence cumulatively suggest the relevance of mtDNA epigenetics to cancer, with some interest already expressed in the use of mtDNA methylation as a biomarker for diagnostic purposes (133, 138). However, possible relationships and applications have yet to be fully explored, especially when we lack basic quantification of methylation across cancer types and stages (139). Filling these gaps in knowledge may assist in reconciling previous discordant evidence among cancer types.

## Conclusion

This review provides a high-level overview of the nature of mtDNA mutations associated with cancer across multiple tumor types. General mutational patterns are placed in the context of evolutionary processes affecting the persistence of these mutations, providing an important foundation for studies of mtDNA and cancer. We provide recommendations for best practices in assessing mtDNA alterations associated with cancer and suggest promising areas for future research.

Alterations to mitochondrial dynamics during tumorigenesis run the full gamut of possibilities, affecting biogenesis, metabolism, and virtually all other aspects of mitochondrial function. As data regarding somatic mtDNA mutations associated with cancer accumulate, it is apparent that some genetic alterations in the mitochondria contribute to tumorigenesis while others simple continue to collect as the cancer progresses. Mutations in nuclear-encoded genes, along with corresponding changes to the cellular function of surrounding cells, may either promote additional changes in mtDNA or provide constraint to what changes may proliferate. Tumors are populations of cells, with each cell possessing its own population of mitochondria, and the fate of mtDNA mutations depend on the effects of selection at multiple levels of complexity. The mtDNA landscape of cancer appears to promote plasticity and adaptability of mitochondria to the ever-changing environment of a tumor. Identifying specific mutations useful as biomarkers, therefore, will require broader sampling of mitochondrial genomes from diverse cancer types at multiple stages of progression, and careful modeling to assess the frequency with which these mutations persist in tumors.

Mitochondria associated with normal and cancer cells represent a microscopic tale of two cities. The genomes of each are subjected to mutation, but cellular and molecular forces differentially shape the fate of such variants. This duality provides the opportunity to inform both basic knowledge of cellular function and translational medicine.

## Author Contributions

KH and SD drafted, edited, and approved the manuscript.

## Conflict of Interest Statement

The authors declare that the research was conducted in the absence of any commercial or financial relationships that could be construed as a potential conflict of interest.

## References

[1] Taylor RW Turnbull DM. Mitochondrial DNA mutations in human disease. Nat Rev Genet (2005) 6(5):389–402. 10.1038/nrg1606 15861210 PMC1762815

[2] Schon EA DiMauro S Hirano M. Human mitochondrial DNA: roles of inherited and somatic mutations. Nat Rev Genet (2012) 13(12):878–90. 10.1038/nrg3275 23154810 PMC3959762

[3] Giampazolias E Tait SW. Mitochondria and the hallmarks of cancer. FEBS J (2016) 283(5):803–14. 10.1111/febs.13603 26607558

[4] Rogalinska M. The role of mitochondria in cancer induction, progression and changes in metabolism. Mini Rev Med Chem (2016) 16(7):524–30. 10.2174/1389557515666151016124331 26471969

[5] Vyas S Zaganjor E Haigis MC. Mitochondria and cancer. Cell (2016) 166(3):555–66. 10.1016/j.cell.2016.07.002 27471965 PMC5036969

[6] Senft D Ronai ZA Regulators of mitochondrial dynamics in cancer. Curr Opin Cell Biol (2016) 39:43–52. 10.1016/j.ceb.2016.02.001 26896558 PMC4828329

[7] Warburg O On the origin of cancer cells. Science (1956) 123(3191):309–14. 10.1126/science.123.3191.309 13298683

[8] Warburg O On respiratory impairment in cancer cells. Science (1956) 124(3215):269–70. 13351639

[9] Zong WX Rabinowitz JD White E. Mitochondria and cancer. Mol Cell (2016) 61(5):667–76. 10.1016/j.molcel.2016.02.011 26942671 PMC4779192

[10] Shidara Y Yamagata K Kanamori T Nakano K Kwong JQ Manfredi G Positive contribution of pathogenic mutations in the mitochondrial genome to the promotion of cancer by prevention from apoptosis. Cancer Res (2005) 65(5):1655–63. 10.1158/0008-5472.CAN-04-2012 15753359

[11] Stefano GB Kream RM Cancer: mitochondrial origins. Med Sci Monitor (2015) 21:3736–9. 10.12659/msm.895990 PMC4671449 26621573

[12] Srinivasan S Guha M Kashina A Avadhani NG Mitochondrial dysfunction and mitochondrial dynamics – the cancer connection. Biochim Biophys Acta (2017) 1858(8):602–14. 10.1016/j.bbabio.2017.01.004 28104365 PMC5487289

[13] Munoz-Gomez SA Wideman JG Roger AJ Slamovits CH. The origin of mitochondrial cristae from alphaproteobacteria. Mol Biol Evol (2017) 34(4):943–56. 10.1093/molbev/msw298 28087774

[14] Allen JF. Why chloroplasts and mitochondria retain their own genomes and genetic systems: colocation for redox regulation of gene expression. Proc Natl Acad Sci U S A (2015) 112(33):10231–8. 10.1073/pnas.1500012112 26286985 PMC4547249

[15] Ju YS Alexandrov LB Gerstung M Martincorena I Nik-Zainal S Ramakrishna M Origins and functional consequences of somatic mitochondrial DNA mutations in human cancer. Elife (2014) 3:02935. 10.7554/eLife.02935 25271376 PMC4371858

[16] van Oven M PhyloTree Build 17: growing the human mitochondrial DNA tree. Forensic Sci Int Genet Suppl Ser (2015) 5:e392–4. 10.1016/j.fsigss.2015.09.155

[17] Wallace DC Shoffner JM Trounce I Brown MD Ballinger SW Corral-Debrinski M Mitochondrial DNA mutations in human degenerative diseases and aging. Biochim Biophys Acta (1995) 1271(1):141–51. 10.1016/0925-4439(95)00021-U 7599200

[18] Krzywinski M Schein J Birol I Connors J Gascoyne R Horsman D Circos: an information aesthetic for comparative genomics. Genome Res (2009) 19(9):1639–45. 10.1101/gr.092759.109 19541911 PMC2752132

[19] Polyak K Li Y Zhu H Lengauer C Willson JKV Markowitz SD Somatic mutations of the mitochondrial genome in human colorectal tumours. Nat Genet (1998) 20(3):291–3. 10.1038/3108 9806551

[20] Stratton MR Campbell PJ Futreal PA The cancer genome. Nature (2009) 458(7239):719–24. 10.1038/nature07943 19360079 PMC2821689

[21] Stewart JB Alaei-Mahabadi B Sabarinathan R Samuelsson T Gorodkin J Gustafsson CM Simultaneous DNA and RNA mapping of somatic mitochondrial mutations across diverse human cancers. PLoS Genet (2015) 11(6):e1005333. 10.1371/journal.pgen.1005333 26125550 PMC4488357

[22] Dasgupta S Hoque MO Upadhyay S Sidransky D Forced cytochrome B gene mutation expression induces mitochondrial proliferation and prevents apoptosis in human uroepithelial SV-HUC-1 cells. Int J Cancer (2009) 125(12):2829–35. 10.1002/ijc.24701 19569044 PMC2988469

[23] Hanahan D Weinberg RA Hallmarks of cancer: the next generation. Cell (2011) 144(5):646–74. 10.1016/j.cell.2011.02.013 21376230

[24] Khrapko K Coller HA Andre PC Li XC Hanekamp JS Thilly WG. Mitochondrial mutational spectra in human cells and tissues. Proc Natl Acad Sci U S A (1997) 94(25):13798–803. 10.1073/pnas.94.25.13798 9391107 PMC28387

[25] Weissensteiner H Pacher D Kloss-Brandstatter A Forer L Specht G Bandelt HJ HaploGrep 2: mitochondrial haplogroup classification in the era of high-throughput sequencing. Nucleic Acids Res (2016) 44(W1):W58–63. 10.1093/nar/gkw233 27084951 PMC4987869

[26] Czarnecka AM Bartnik E. The role of the mitochondrial genome in ageing and carcinogenesis. J Aging Res (2011) 2011:136435. 10.4061/2011/136435 21403887 PMC3042732

[27] Lam ET Bracci PM Holly EA Chu C Poon A Wan E Mitochondrial DNA sequence variation and risk of pancreatic cancer. Cancer Res (2012) 72(3):686–95. 10.1158/0008-5472.CAN-11-1682 22174369 PMC3271167

[28] Blein S Bardel C Danjean V McGuffog L Healey S Barrowdale D An original phylogenetic approach identified mitochondrial haplogroup T1a1 as inversely associated with breast cancer risk in BRCA2 mutation carriers. Breast Cancer Res (2015) 17:61. 10.1186/s13058-015-0567-2 25925750 PMC4478717

[29] Salgado J Honorato B Garcia-Foncillas J Review: mitochondrial defects in breast cancer. Clin Med Oncol (2008) 2:199–207. 10.4137/CMO.S524 21892280 PMC3161697

[30] Galtier N Enard D Radondy Y Bazin E Belkhir K. Mutation hot spots in mammalian mitochondrial DNA. Genome Res (2006) 16(2):215–22. 10.1101/gr.4305906 16354751 PMC1361717

[31] McMahon S LaFramboise T. Mutational patterns in the breast cancer mitochondrial genome, with clinical correlates. Carcinogenesis (2014) 35(5):1046–54. 10.1093/carcin/bgu012 24442641 PMC4004206

[32] Kloss-Brandstatter A Weissensteiner H Erhart G Schafer G Forer L Schonherr S Validation of next-generation sequencing of entire mitochondrial genomes and the diversity of mitochondrial DNA mutations in oral squamous cell carcinoma. PLoS One (2015) 10(8):e0135643. 10.1371/journal.pone.0135643 26262956 PMC4532422

[33] Larman TC DePalma SR Hadjipanayis AG Cancer Genome Atlas Research N Protopopov A Zhang J Spectrum of somatic mitochondrial mutations in five cancers. Proc Natl Acad Sci U S A (2012) 109(35):14087–91. 10.1073/pnas.1211502109 22891333 PMC3435197

[34] Alexandrov LB Nik-Zainal S Wedge DC Aparicio SA Behjati S Biankin AV Signatures of mutational processes in human cancer. Nature (2013) 500(7463):415–21. 10.1038/nature12477 23945592 PMC3776390

[35] Lee HC Huang KH Yeh TS Chi CW. Somatic alterations in mitochondrial DNA and mitochondrial dysfunction in gastric cancer progression. World J Gastroenterol (2014) 20(14):3950–9. 10.3748/wjg.v20.i14.3950 24744584 PMC3983450

[36] Kurelac I MacKay A Lambros MB Di Cesare E Cenacchi G Ceccarelli C Somatic complex I disruptive mitochondrial DNA mutations are modifiers of tumorigenesis that correlate with low genomic instability in pituitary adenomas. Hum Mol Genet (2013) 22(2):226–38. 10.1093/hmg/dds422 23049073

[37] Shen L Wei J Chen T He J Qu J He X Evaluating mitochondrial DNA in patients with breast cancer and benign breast disease. J Cancer Res Clin Oncol (2011) 137(4):669–75. 10.1007/s00432-010-0912-x 20552226 PMC11827960

[38] Iommarini L Kurelac I Capristo M Calvaruso MA Giorgio V Bergamini C Different mtDNA mutations modify tumor progression in dependence of the degree of respiratory complex I impairment. Hum Mol Genet (2014) 23(6):1453–66. 10.1093/hmg/ddt533 24163135

[39] Dasgupta S Shao C Keane TE Duberow DP Mathies RA Fisher PB Detection of mitochondrial deoxyribonucleic acid alterations in urine from urothelial cell carcinoma patients. Int J Cancer (2012) 131(1):158–64. 10.1002/ijc.26357 21826645 PMC3328657

[40] Dasgupta S Hoque MO Upadhyay S Sidransky D. Mitochondrial cytochrome B gene mutation promotes tumor growth in bladder cancer. Cancer Res (2008) 68(3):700–6. 10.1158/0008-5472.CAN-07-5532 18245469

[41] Namslauer I Brzezinski P. A mitochondrial DNA mutation linked to colon cancer results in proton leaks in cytochrome c oxidase. Proc Natl Acad Sci U S A (2009) 106(9):3402–7. 10.1073/pnas.0811450106 19218458 PMC2651238

[42] Ghaffarpour M Mahdian R Fereidooni F Kamalidehghan B Moazami N Houshmand M The mitochondrial ATPase6 gene is more susceptible to mutation than the ATPase8 gene in breast cancer patients. Cancer Cell Int (2014) 14(1):21 10.1186/1475-2867-14-21 24588805 PMC3942513

[43] Fliss MS Usadel H Caballero OL Wu L Buta MR Eleff SM Facile detection of mitochondrial DNA mutations in tumors and bodily fluids. Science (2000) 287(5460):2017–9. 10.1126/science.287.5460.2017 10720328

[44] Sanchez-Cespedes M Parrella P Nomoto S Cohen D Xiao Y Esteller M Identification of a mononucleotide repeat as a major target for mitochondrial DNA alterations in human tumors. Cancer Res (2001) 61(19):7015–9. 11585726

[45] Duberow DP Brait M Hoque MO Theodorescu D Sidransky D Dasgupta S High-performance detection of somatic D-loop mutation in urothelial cell carcinoma patients by polymorphism ratio sequencing. J Mol Med (Berl) (2016) 94(9):1015–24. 10.1007/s00109-016-1407-2 27030170 PMC8883517

[46] Yadav N Chandra D. Mitochondrial DNA mutations and breast tumorigenesis. Biochim Biophys Acta (2013) 1836(2):336–44. 10.1016/j.bbcan.2013.10.002 24140413 PMC3891589

[47] Masuda S Kadowaki T Kumaki N Tang X Tokuda Y Yoshimura S Analysis of gene alterations of mitochondrial DNA D-loop regions to determine breast cancer clonality. Br J Cancer (2012) 107(12):2016–23. 10.1038/bjc.2012.505 23169290 PMC3516690

[48] Yu Y Lv F Lin H Qian G Jiang YS Pang LX Mitochondrial ND3 G10398A mutation: a biomarker for breast cancer. Genet Mol Res (2015) 14(4):17426–31. 10.4238/2015.December.21.12 26782384

[49] Skonieczna K Malyarchuk BA Grzybowski T. The landscape of mitochondrial DNA variation in human colorectal cancer on the background of phylogenetic knowledge. Biochim Biophys Acta (2012) 1825(2):153–9. 10.1016/j.bbcan.2011.11.004 22178219

[50] Lievre A Blons H Houllier AM Laccourreye O Brasnu D Beaune P Clinicopathological significance of mitochondrial D-Loop mutations in head and neck carcinoma. Br J Cancer (2006) 94(5):692–7. 10.1038/sj.bjc.6602993 16495928 PMC2361200

[51] Dasgupta S Koch R Westra WH Califano JA Ha PK Sidransky D Mitochondrial DNA mutation in normal margins and tumors of recurrent head and neck squamous cell carcinoma patients. Cancer Prev Res (Phila) (2010) 3(9):1205–11. 10.1158/1940-6207.CAPR-10-0018 20660573 PMC3040952

[52] Matsuyama W Nakagawa M Wakimoto J Hirotsu Y Kawabata M Osame M Mitochondrial DNA mutation correlates with stage progression and prognosis in non-small cell lung cancer. Hum Mutat (2003) 21(4):441–3. 10.1002/humu.10196 12655558

[53] Suzuki M Toyooka S Miyajima K Iizasa T Fujisawa T Bekele NB Alterations in the mitochondrial displacement loop in lung cancers. Clin Cancer Res (2003) 9(15):5636–41. 14654546

[54] Lee HC Yin PH Lin JC Wu CC Chen CY Wu CW Mitochondrial genome instability and mtDNA depletion in human cancers. Ann N Y Acad Sci (2005) 1042:109–22. 10.1196/annals.1338.011 15965052

[55] Dasgupta S Yung RC Westra WH Rini DA Brandes J Sidransky D Following mitochondrial footprints through a long mucosal path to lung cancer. PLoS One (2009) 4(8):e6533 10.1371/journal.pone.0006533 19657397 PMC2719062

[56] Dasgupta S Soudry E Mukhopadhyay N Shao C Yee J Lam S Mitochondrial DNA mutations in respiratory complex-I in never-smoker lung cancer patients contribute to lung cancer progression and associated with EGFR gene mutation. J Cell Physiol (2012) 227(6):2451–60. 10.1002/jcp.22980 21830212 PMC3256258

[57] Liu VW Shi HH Cheung AN Chiu PM Leung TW Nagley P High incidence of somatic mitochondrial DNA mutations in human ovarian carcinomas. Cancer Res (2001) 61(16):5998–6001. 11507041

[58] Bragoszewski P Kupryjanczyk J Bartnik E Rachinger A Ostrowski J. Limited clinical relevance of mitochondrial DNA mutation and gene expression analyses in ovarian cancer. BMC Cancer (2008) 8:292. 10.1186/1471-2407-8-292 18842121 PMC2571110

[59] Guerra F Perrone AM Kurelac I Santini D Ceccarelli C Cricca M Mitochondrial DNA mutation in serous ovarian cancer: implications for mitochondria-coded genes in chemoresistance. J Clin Oncol (2012) 30(36):e373–8. 10.1200/JCO.2012.43.5933 23150702

[60] Petros JA Baumann AK Ruiz-Pesini E Amin MB Sun CQ Hall J mtDNA mutations increase tumorigenicity in prostate cancer. Proc Natl Acad Sci U S A (2005) 102(3):719–24. 10.1073/pnas.0408894102 15647368 PMC545582

[61] Kloss-Brandstatter A Schafer G Erhart G Huttenhofer A Coassin S Seifarth C Somatic mutations throughout the entire mitochondrial genome are associated with elevated PSA levels in prostate cancer patients. Am J Hum Genet (2010) 87(6):802–12. 10.1016/j.ajhg.2010.11.001 21129724 PMC2997360

[62] Arnold RS Fedewa SA Goodman M Osunkoya AO Kissick HT Morrissey C Bone metastasis in prostate cancer: recurring mitochondrial DNA mutation reveals selective pressure exerted by the bone microenvironment. Bone (2015) 78:81–6. 10.1016/j.bone.2015.04.046 25952970 PMC4466124

[63] Philley JV Kannan A Qin W Sauter ER Ikebe M Hertweck KL Complex-I alteration and enhanced mitochondrial fusion are associated with prostate cancer progression. J Cell Physiol (2016) 231(6):1364–74. 10.1002/jcp.25240 26530043 PMC5741292

[64] Stoneking M. Hypervariable sites in the mtDNA control region are mutational hotspots. Am J Hum Genet (2000) 67(4):1029–32. 10.1086/303092 10968778 PMC1287875

[65] Nicholls TJ Minczuk M. In D-loop: 40 years of mitochondrial 7S DNA. Exp Gerontol (2014) 56:175–81. 10.1016/j.exger.2014.03.027 24709344

[66] Kuo SJ Chen M Ma GC Chen ST Chang SP Lin WY Number of somatic mutations in the mitochondrial D-loop region indicates poor prognosis in breast cancer, independent of TP53 mutation. Cancer Genet Cytogenet (2010) 201(2):94–101. 10.1016/j.cancergencyto.2010.05.013 20682393

[67] Akouchekian M Houshmand M Hemati S Ansaripour M Shafa M. High rate of mutation in mitochondrial DNA displacement loop region in human colorectal cancer. Dis Colon Rectum (2009) 52(3):526–30. 10.1007/DCR.0b013e31819acb99 19333057

[68] Vogelstein B Papadopoulos N Velculescu VE Zhou S Diaz LA Jr Kinzler KW. Cancer genome landscapes. Science (2013) 339(6127):1546–58. 10.1126/science.1235122 23539594 PMC3749880

[69] Seyfried TN. Cancer as a mitochondrial metabolic disease. Front Cell Dev Biol (2015) 3:43. 10.3389/fcell.2015.00043 26217661 PMC4493566

[70] Melvin RG Ballard JWO. Cellular and population level processes influence the rate, accumulation and observed frequency of inherited and somatic mtDNA mutations. Mutagenesis (2017) 32(3):323–34. 10.1093/mutage/gex004 28521046

[71] Stafford P Chen-Quin EB. The pattern of natural selection in somatic cancer mutations of human mtDNA. J Hum Genet (2010) 55(9):605–12. 10.1038/jhg.2010.76 20613764

[72] Zhidkov I Livneh EA Rubin E Mishmar D. MtDNA mutation pattern in tumors and human evolution are shaped by similar selective constraints. Genome Res (2009) 19(4):576–80. 10.1101/gr.086462.108 19211544 PMC2665776

[73] Li M Schroder R Ni S Madea B Stoneking M. Extensive tissue-related and allele-related mtDNA heteroplasmy suggests positive selection for somatic mutations. Proc Natl Acad Sci U S A (2015) 112(8):2491–6. 10.1073/pnas.1419651112 25675502 PMC4345623

[74] Pereira L Soares P Máximo V Samuels DC. Somatic mitochondrial DNA mutations in cancer escape purifying selection and high pathogenicity mutations lead to the oncocytic phenotype: pathogenicity analysis of reported somatic mtDNA mutations in tumors. BMC Cancer (2012) 12(1):53. 10.1186/1471-2407-12-53 22299657 PMC3342922

[75] Damas J Samuels DC Carneiro J Amorim A Pereira F Mitochondrial DNA rearrangements in health and disease – a comprehensive study. Hum Mutat (2014) 35(1):1–14. 10.1002/humu.22452 24115352

[76] Dimberg J Hong TT Nguyen LT Skarstedt M Lofgren S Matussek A Common 4977 bp deletion and novel alterations in mitochondrial DNA in Vietnamese patients with breast cancer. Springerplus (2015) 4:58 10.1186/s40064-015-0843-8 25674508 PMC4318828

[77] Chen T He J Shen L Fang H Nie H Jin T The mitochondrial DNA 4,977-bp deletion and its implication in copy number alteration in colorectal cancer. BMC Med Genet (2011) 12:8. 10.1186/1471-2350-12-8 21232124 PMC3025938

[78] Dimberg J Hong TT Skarstedt M Lofgren S Zar N Matussek A. Novel and differential accumulation of mitochondrial DNA deletions in Swedish and vietnamese patients with colorectal cancer. Anticancer Res (2014) 34(1):147–52. 10.1186/s40064-015-0843-8 24403455

[79] Datta S Chattopadhyay E Ray JG Majumder M Roy PD Roy B D-loop somatic mutations and approximately 5 kb “common” deletion in mitochondrial DNA: important molecular markers to distinguish oral precancer and cancer. Tumour Biol (2015) 36(4):3025–33. 10.1007/s13277-014-2937-2 25527154

[80] Kim TM Laird PW Park PJ. The landscape of microsatellite instability in colorectal and endometrial cancer genomes. Cell (2013) 155(4):858–68. 10.1016/j.cell.2013.10.015 24209623 PMC3871995

[81] Cortes-Ciriano I Lee S Park WY Kim TM Park PJ. A molecular portrait of microsatellite instability across multiple cancers. Nat Commun (2017) 8:15180. 10.1038/ncomms15180 28585546 PMC5467167

[82] Hiyama T Tanaka S Shima H Kose K Tuncel H Ito M Somatic mutation in mitochondrial DNA and nuclear microsatellite instability in gastric cancer. Oncol Rep (2003) 10(6):1837–41. 10.3892/or.10.6.1837 14534705

[83] Parrella P Seripa D Matera MG Rabitti C Rinaldi M Mazzarelli P Mutations of the D310 mitochondrial mononucleotide repeat in primary tumors and cytological specimens. Cancer Lett (2003) 190(1):73–7. 10.1016/S0304-3835(02)00578-5 12536079

[84] Pavicic WH Laguens M Richard SM. Analysis association between mitochondrial genome instability and xenobiotic metabolizing genes in human breast cancer. Mol Med (2009) 15(5–6):160–5. 10.2119/molmed.2008.00136 19287511 PMC2654850

[85] Burgart LJ Zheng J Shu Q Strickler JG Shibata D. Somatic mitochondrial mutation in gastric cancer. Am J Pathol (1995) 147(4):1105–11. 7573355 PMC1871018

[86] Nie H Shu H Vartak R Milstein AC Mo Y Hu X Mitochondrial common deletion, a potential biomarker for cancer occurrence, is selected against in cancer background: a meta-analysis of 38 studies. PLoS One (2013) 8(7):e67953. 10.1371/journal.pone.0067953 23861839 PMC3701633

[87] Ju YS Tubio JM Mifsud W Fu B Davies HR Ramakrishna M Frequent somatic transfer of mitochondrial DNA into the nuclear genome of human cancer cells. Genome Res (2015) 25(6):814–24. 10.1101/gr.190470.115 25963125 PMC4448678

[88] Reznik E Miller ML Şenbabaoğlu Y Riaz N Sarungbam J Tickoo SK Mitochondrial DNA copy number variation across human cancers. Elife (2016) 5:e10769. 10.7554/eLife.10769 26901439 PMC4775221

[89] Savagner F Franc B Guyetant S Rodien P Reynier P Malthiery Y. Defective mitochondrial ATP synthesis in oxyphilic thyroid tumors. J Clin Endocrinol Metab (2001) 86(10):4920–5. 10.1210/jcem.86.10.7894 11600563

[90] Lynch SM Weinstein SJ Virtamo J Lan Q Liu CS Cheng WL Mitochondrial DNA copy number and pancreatic cancer in the alpha-tocopherol beta-carotene cancer prevention study. Cancer Prev Res (Phila) (2011) 4(11):1912–9. 10.1158/1940-6207.CAPR-11-0002 21859925 PMC3208722

[91] Lee HC Li SH Lin JC Wu CC Yeh DC Wei YH. Somatic mutations in the D-loop and decrease in the copy number of mitochondrial DNA in human hepatocellular carcinoma. Mutat Res (2004) 547(1–2):71–8. 10.1016/j.mrfmmm.2003.12.011 15013701

[92] Thyagarajan B Wang R Barcelo H Koh W-P Yuan J-M. Mitochondrial copy number is associated with colorectal cancer risk. Cancer Epidemiol Biomarkers Prev (2012) 21(9):1574–81. 10.1158/1055-9965.EPI-12-0138-T 22787200 PMC3437007

[93] Mizumachi T Muskhelishvili L Naito A Furusawa J Fan CY Siegel ER Increased distributional variance of mitochondrial DNA content associated with prostate cancer cells as compared with normal prostate cells. Prostate (2008) 68(4):408–17. 10.1002/pros.20697 18196528 PMC2268637

[94] Payne BA Wilson IJ Yu-Wai-Man P Coxhead J Deehan D Horvath R Universal heteroplasmy of human mitochondrial DNA. Hum Mol Genet (2013) 22(2):384–90. 10.1093/hmg/dds435 23077218 PMC3526165

[95] He Y Wu J Dressman DC Iacobuzio-Donahue C Markowitz SD Velculescu VE Heteroplasmic mitochondrial DNA mutations in normal and tumour cells. Nature (2010) 464(7288):610–4. 10.1038/nature08802 20200521 PMC3176451

[96] Stefano GB Kream RM Mitochondrial DNA heteroplasmy in human health and disease. Biomed Rep (2016) 4(3):259–62. 10.3892/br.2016.590 26998260 PMC4774312

[97] Arnold RS Sun Q Sun CQ Richards JC O’Hearn S Osunkoya AO An inherited heteroplasmic mutation in mitochondrial gene COI in a patient with prostate cancer alters reactive oxygen, reactive nitrogen and proliferation. Biomed Res Int (2013) 2013:239257. 10.1155/2013/239257 23509693 PMC3591245

[98] Stewart JB Chinnery PF. The dynamics of mitochondrial DNA heteroplasmy: implications for human health and disease. Nat Rev Genet (2015) 16(9):530–42. 10.1038/nrg3966 26281784

[99] Coller HA Khrapko K Bodyak ND Nekhaeva E Herrero-Jimenez P Thilly WG. High frequency of homoplasmic mitochondrial DNA mutations in human tumors can be explained without selection. Nat Genet (2001) 28(2):147–50. 10.1038/88859 11381261

[100] Pagliarini DJ Calvo SE Chang B Sheth SA Vafai SB Ong SE A mitochondrial protein compendium elucidates complex I disease biology. Cell (2008) 134(1):112–23. 10.1016/j.cell.2008.06.016 18614015 PMC2778844

[101] Guo J Zheng L Liu W Wang X Wang Z Wang Z Frequent truncating mutation of TFAM induces mitochondrial DNA depletion and apoptotic resistance in microsatellite-unstable colorectal cancer. Cancer Res (2011) 71(8):2978–87. 10.1158/0008-5472.CAN-10-3482 21467167 PMC3710668

[102] Linkowska K Jawien A Marszalek A Malyarchuk BA Tonska K Bartnik E Mitochondrial DNA polymerase gamma mutations and their implications in mtDNA alterations in colorectal cancer. Ann Hum Genet (2015) 79(5):320–8. 10.1111/ahg.12111 25850945

[103] van Gisbergen MW Voets AM Starmans MH de Coo IF Yadak R Hoffmann RF How do changes in the mtDNA and mitochondrial dysfunction influence cancer and cancer therapy? Challenges, opportunities and models. Mutat Res Rev Mutat Res (2015) 764:16–30. 10.1016/j.mrrev.2015.01.001 26041263

[104] Kamp WM Wang PY Hwang PM. TP53 mutation, mitochondria and cancer. Curr Opin Genet Dev (2016) 38:16–22. 10.1016/j.gde.2016.02.007 27003724 PMC5028230

[105] Lee HC Hsu LS Yin PH Lee LM Chi CW. Heteroplasmic mutation of mitochondrial DNA D-loop and 4977-bp deletion in human cancer cells during mitochondrial DNA depletion. Mitochondrion (2007) 7(1–2):157–63. 10.1016/j.mito.2006.11.016 17280876

[106] Kaipparettu BA Ma Y Park JH Lee TL Zhang Y Yotnda P Crosstalk from non-cancerous mitochondria can inhibit tumor properties of metastatic cells by suppressing oncogenic pathways. PLoS One (2013) 8(5):e61747. 10.1371/journal.pone.0061747 23671572 PMC3650012

[107] Kirches E. MtDNA as a cancer marker: a finally closed chapter? Curr Genomics (2017) 18(3):255–67. 10.2174/1389202918666170105093635 28659721 PMC5476953

[108] Hibi K Nakayama H Yamazaki T Takase T Taguchi M Kasai Y Detection of mitochondrial DNA alterations in primary tumors and corresponding serum of colorectal cancer patients. Int J Cancer (2001) 94(3):429–31. 10.1002/ijc.1480 11745425

[109] Parrella P Xiao Y Fliss M Sanchez-Cespedes M Mazzarelli P Rinaldi M Detection of mitochondrial DNA mutations in primary breast cancer and fine-needle aspirates. Cancer Res (2001) 61(20):7623–6. 11606403

[110] Kim A Mitochondrial DNA somatic mutation in cancer. Toxicol Res (2014) 30(4):235–42. 10.5487/TR.2014.30.4.235 25584142 PMC4289923

[111] Zhang X de Milito A Olofsson MH Gullbo J D’Arcy P Linder S. Targeting mitochondrial function to treat quiescent tumor cells in solid tumors. Int J Mol Sci (2015) 16(11):27313–26. 10.3390/ijms161126020 26580606 PMC4661878

[112] Caino MC Altieri DC. Molecular pathways: mitochondrial reprogramming in tumor progression and therapy. Clin Cancer Res (2016) 22(3):540–5. 10.1158/1078-0432.CCR-15-0460 26660517 PMC4738153

[113] Indran IR Tufo G Pervaiz S Brenner C. Recent advances in apoptosis, mitochondria and drug resistance in cancer cells. Biochim Biophys Acta (2011) 1807(6):735–45. 10.1016/j.bbabio.2011.03.010 21453675

[114] Lievre A Chapusot C Bouvier AM Zinzindohoue F Piard F Roignot P Clinical value of mitochondrial mutations in colorectal cancer. J Clin Oncol (2005) 23(15):3517–25. 10.1200/JCO.2005.07.044 15908662

[115] Weerts MJ Hollestelle A Sieuwerts AM Foekens JA Sleijfer S Martens JW. Low tumor mitochondrial DNA content is associated with better outcome in breast cancer patients receiving anthracycline-based chemotherapy. Clin Cancer Res (2017) 23(16):4735–43. 10.1158/1078-0432.CCR-17-0032 28420722

[116] Bandelt HJ Kivisild T Parik J Villems R Bravi C Yao YG Lab-specific mutation processes. In: Bandelt HJ Macaulay V Richards M editors. Human Mitochondrial DNA and the Evolution of Homo sapiens, Vol 18 Berlin, Heidelberg: Springer (2006).

[117] Fang H Lu J Wei J Shen LJ Ding Z Li H Mitochondrial DNA mutations in the D-loop region may not be frequent in cervical cancer: a discussion on pitfalls in mitochondrial DNA studies. J Cancer Res Clin Oncol (2009) 135(4):649–51. 10.1007/s00432-008-0542-8 19142663 PMC4710170

[118] Skonieczna K Malyarchuk B Jawien A Marszalek A Banaszkiewicz Z Jarmocik P Heteroplasmic substitutions in the entire mitochondrial genomes of human colon cells detected by ultra-deep 454 sequencing. Forensic Sci Int Genet (2015) 15:16–20. 10.1016/j.fsigen.2014.10.021 25465762

[119] Vidone M Clima R Santorsola M Calabrese C Girolimetti G Kurelac I A comprehensive characterization of mitochondrial DNA mutations in glioblastoma multiforme. Int J Biochem Cell Biol (2015) 63:46–54. 10.1016/j.biocel.2015.01.027 25668474

[120] Just RS Irwin JA Parson W. Mitochondrial DNA heteroplasmy in the emerging field of massively parallel sequencing. Forensic Sci Int Genet (2015) 18:131–9. 10.1016/j.fsigen.2015.05.003 26009256 PMC4550493

[121] Bandelt HJ Yao YG Bravi CM Salas A Kivisild T. Median network analysis of defectively sequenced entire mitochondrial genomes from early and contemporary disease studies. J Hum Genet (2009) 54(3):174–81. 10.1038/jhg.2009.9 19322152

[122] Salas A Yao YG Macaulay V Vega A Carracedo A Bandelt HJ. A critical reassessment of the role of mitochondria in tumorigenesis. PLoS Med (2005) 2(11):e296. 10.1371/journal.pmed.0020296 16187796 PMC1240051

[123] Li H Liu D Lu J Bai Y. Physiology and pathophysiology of mitochondrial DNA. Adv Exp Med Biol (2012) 942:39–51. 10.1007/978-94-007-2869-1_2 22399417 PMC4706180

[124] Bandelt H-J Kloss-Brandstatter A Richards MB Yao Y-G Logan I. The case for the continuing use of the revised Cambridge Reference Sequence (rCRS) and the standardization of notation in human mitochondrial DNA studies. J Hum Genet (2014) 59(2):66–77. 10.1038/jhg.2013.120 24304692

[125] Brandon M Baldi P Wallace DC Mitochondrial mutations in cancer. Oncogene (2006) 25(34):4647–62. 10.1038/sj.onc.1209607 16892079

[126] Covarrubias D Bai RK Wong LJ Leal SM. Mitochondrial DNA variant interactions modify breast cancer risk. J Hum Genet (2008) 53(10):924–8. 10.1007/s10038-008-0331-x 18709563 PMC2767522

[127] Salas A Garcia-Magarinos M Logan I Bandelt HJ. The saga of the many studies wrongly associating mitochondrial DNA with breast cancer. BMC Cancer (2014) 14:659. 10.1186/1471-2407-14-659 25199876 PMC4180149

[128] Kulawiec M Safina A Desouki MM Still I Matsui S Bakin A Tumorigenic transformation of human breast epithelial cells induced by mitochondrial DNA depletion. Cancer Biol Ther (2008) 7(11):1732–43. 10.4161/cbt.7.11.6729 19151587 PMC2783327

[129] Ishikawa K Hashizume O Koshikawa N Fukuda S Nakada K Takenaga K Enhanced glycolysis induced by mtDNA mutations does not regulate metastasis. FEBS Lett (2008) 582(23–24):3525–30. 10.1016/j.febslet.2008.09.024 18805414

[130] Singh KK Kulawiec M Mitochondrial DNA polymorphism and risk of cancer. Methods Mol Biol (2009) 471:291–303. 10.1007/978-1-59745-416-2_15 19109786 PMC2825891

[131] Roberts SA Gordenin DA. Hypermutation in human cancer genomes: footprints and mechanisms. Nat Rev Cancer (2014) 14(12):786–800. 10.1038/nrc3816 25568919 PMC4280484

[132] Alexandrov LB Nik-Zainal S Wedge DC Campbell PJ Stratton MR Deciphering signatures of mutational processes operative in human cancer. Cell Rep (2013) 3(1):246–59. 10.1016/j.celrep.2012.12.008 23318258 PMC3588146

[133] Ghosh S Singh KK Sengupta S Scaria V. Mitoepigenetics: the different shades of grey. Mitochondrion (2015) 25:60–6. 10.1016/j.mito.2015.09.003 26437363

[134] Ghosh S Sengupta S Scaria V Comparative analysis of human mitochondrial methylomes shows distinct patterns of epigenetic regulation in mitochondria. Mitochondrion (2014) 18:58–62. 10.1016/j.mito.2014.07.007 25058022

[135] Esteller M. Cancer epigenomics: DNA methylomes and histone-modification maps. Nat Rev Genet (2007) 8(4):286–98. 10.1038/nrg2005 17339880

[136] Guantes R Rastrojo A Neves R Lima A Aguado B Iborra FJ. Global variability in gene expression and alternative splicing is modulated by mitochondrial content. Genome Res (2015) 25(5):633–44. 10.1101/gr.178426.114 25800673 PMC4417112

[137] Minocherhomji S Tollefsbol TO Singh KK. Mitochondrial regulation of epigenetics and its role in human diseases. Epigenetics (2012) 7(4):326–34. 10.4161/epi.19547 22419065 PMC3368816

[138] Iacobazzi V Castegna A Infantino V Andria G. Mitochondrial DNA methylation as a next-generation biomarker and diagnostic tool. Mol Genet Metab (2013) 110(1–2):25–34. 10.1016/j.ymgme.2013.07.012 23920043

[139] Ferreira A Serafim TL Sardao VA Cunha-Oliveira T. Role of mtDNA-related mitoepigenetic phenomena in cancer. Eur J Clin Invest (2015) 45(Suppl 1):44–9. 10.1111/eci.12359 25524586

